# Dihydromyricetin Alleviated Acetaminophen-Induced Acute Kidney Injury via Nrf2-Dependent Anti-Oxidative and Anti-Inflammatory Effects

**DOI:** 10.3390/ijms26052365

**Published:** 2025-03-06

**Authors:** Jianan Shi, Xiufang Peng, Junyi Huang, Mengyi Zhang, Yuqin Wang

**Affiliations:** Department of Pharmacology, School of Pharmacy, Nantong University, Nantong 226019, China; sjn1234562021@163.com (J.S.); 18286607097@163.com (X.P.); hhhhjy99@163.com (J.H.); 15250731769@139.com (M.Z.)

**Keywords:** dihydromyricetin, acetaminophen, acute kidney injury, Nrf2

## Abstract

Acute kidney injury (AKI) is a common side effect of acetaminophen (APAP) overdose. Dihydromyricetin (DHM) is the most abundant flavonoid in rattan tea, which has a wide range of pharmacological effects. In the current study, APAP-induced AKI models were established both in vivo and in vitro. The results showed that DHM pretreatment remarkably alleviated APAP-induced AKI by promoting antioxidant capacity through the nuclear factor erythroid-related factor 2 (Nrf2) signaling pathway in vivo. In addition, DHM reduced ROS production and mitochondrial dysfunction, thereby alleviating APAP-induced cytotoxicity in HK-2 cells. The way in which DHM improved the antioxidant capacity of HK-2 cells was through promoting the activation of the Nrf2-mediated pathway and inhibiting the expression levels of inflammation-related proteins. Furthermore, Nrf2 siRNA partially canceled out the protective effect of DHM against the cytotoxicity caused by APAP in HK-2 cells. Altogether, the protective effect of DHM on APAP-induced nephrotoxicity was related to Nrf2-dependent antioxidant and anti-inflammatory effects.

## 1. Introduction

Acetaminophen (APAP) is a widely used over-the-counter drug, and toxicities induced by an excess of APAP commonly occur in clinics [1,2]. Excessive APAP-induced acute kidney injury (AKI) appears in approximately 1–2% of patients, and sometimes AKI occurs simultaneously with APAP-induced hepatotoxicity [3,4].

A great deal of research has revealed that oxidative stress accelerates the progression of various kidney diseases, including AKI [5]. Superfluous N-acetyl-P-benzoquinone imine (NAPQI) produced by excessive APAP consumes cellular glutathione (GSH), while the unbound NAQPI covalently binds to cellular macromolecules and lipids, and then induces lipid peroxidation, mitochondrial dysfunction, and DNA damage [6]. Mitochondrial dysfunction further promotes ROS generation and exacerbates cell damage and renal dysfunction [1,7]. Nuclear factor erythroid-related factor 2 (Nrf2) is an important member of the antioxidant system in vivo. It is physiologically bound to its inhibitor Kelch, like ECH-associated protein 1 (keap1) in cytoplasm, and only slightly activates in the nucleus to maintain cell homeostasis [8]. Under oxidative stress, Nrf2 is released from the Nrf2-Keap1 complex after the phosphorylation of Ser-40, and transports to the nucleus to encode detoxification enzymes and sulfur oxidase, such as Heme Oxygenase-1 (HO-1) and NAD (P) H: NADH Quinone Oxidoreductase 1 (NQO1), etc. [9]. Hence, the activation of Nrf2 during the oxidative stress induced by drugs and their toxic metabolites effectively alleviates oxidative stress-triggered damage [10,11,12].

Besides excessive ROS, the overproduction of NAPQI also causes the release of high-mobility group box 1 (HMGB1), which has pro-inflammatory properties [13,14]. Upon the binding of HMGB1 to toll-like receptor 4 (TLR4), nuclear factor kappa B (NF-κB) is activated and then promotes the formation of NOD-like receptor protein 3 (NLRP3) inflammasome, by regulating the transcription of NF-κB-dependent genes such as Nlrp3, Il1β, and Il18 [15]. Recently, it has been found that the activation of NLRP3 inflammasome is also a key mediator in the APAP-induced inflammatory response [15,16]. In addition, excessive ROS have been reported to promote the transfer of NF-κB p65 into the nucleus by modifying the phosphorylation of IκB; meanwhile, NF-κB p65 can up-regulate antioxidant proteins, such as HO-1 [17,18]. Therefore, the inhibition of the NF-κB signaling pathway has potential as a therapeutic intervention for APAP-induced nephrotoxicity.

Dihydromyricetin (DHM) is the most abundant flavonoid in rattan tea, which displays a broad range of pharmacological effects [19,20]. For instance, DHM could inhibit the activation of the NF-κB signaling pathway and resist inflammation [21]. Recently, DHM was found to significantly repress the TGF-β 1-induced up-regulation of miR-34a and renal fibrosis [22]. It has also been shown that DHM is beneficial for the amelioration of atherosclerosis and diabetic cardiomyopathy [23,24]. Herein, we explored the potential effects and possible mechanisms of DHM on APAP-induced AKI. Our results demonstrated that DHM alleviated APAP-induced AKI both in vivo and in vitro, by up-regulating the Nrf2-mediated signaling pathway and inhibiting renal inflammation. Together, these findings may provide new insights into possible applications of DHM for APAP-induced AKI.

## 2. Results

### 2.1. DHM Pretreatment Alleviated APAP-Induced AKI in Mice

**Figure 1:**
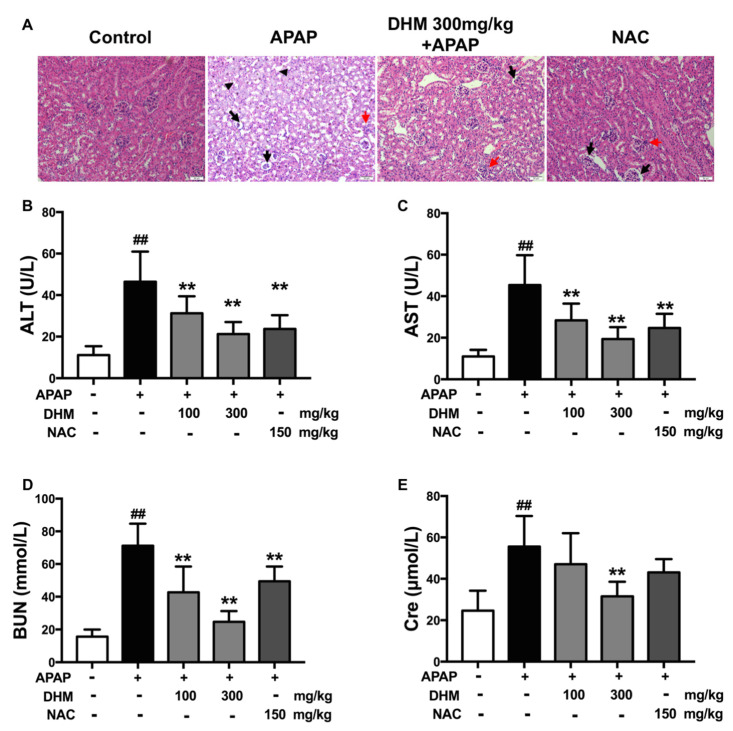
DHM pretreatment alleviated APAP-induced AKI in mice. The C57BL/6J mice were administered with either DHM or saline for 7 continuous days, and then intraperitoneally injected with 500 mg/kg APAP to induce AKI. Twenty-four hours after APAP injection, kidneys and plasma were collected for subsequent experiments. (**A**) The representative images of H&E staining in different groups were presented (Scale = 50 μm, black arrow, glomerular atrophy; black triangle, vacuolation; and red arrow, infiltration). Statistical diagram of serum ALT (**B**), AST (**C**), BUN (**D**), and Cre (**E**) levels in different groups. The results are mean ± SD. The data were obtained from at least six mice. ^##^ *p* < 0.01 vs. control mice; ** *p* < 0.01 vs. the APAP-treated mice.

Initially, we confirmed the protective effects of DHM against APAP-induced AKI in C57/BL6J mice. The induction of nephrotoxicity in mice was confirmed based on H&E staining and the elevated serum levels of ALT, AST, BUN, and Cre at 24 h following APAP treatment. As shown in Figure 1A, an overdose of APAP caused renal damage such as glomerular atrophy, tubular vacuolation, and lymphocyte infiltration, while DHM pretreatment significantly alleviated the renal pathological damage induced by APAP. The serum levels of AST, ALT, BUN, and Cre markedly increased in the APAP-treated group, and DHM pretreatment significantly inhibited an APAP-induced increase in a dose-dependent manner (Figure 1B–E). A point worth making is that although NAC has a significant inhibitory effect on elevated AST, ALT, and BUN levels, its inhibitory effect on Cre was less pronounced, which suggests that its influence on APAP-induced nephrotoxicity may not be the same as its effect on APAP-induced hepatotoxicity, as previously reported [25]. These data together suggest that DHM preconditioning could significantly alleviate APAP-induced AKI in vivo.

### 2.2. DHM Pretreatment Alleviated APAP-Induced Oxidative Stress in Renal Tissue Through Nrf2 Signaling Pathway

**Figure 2:**
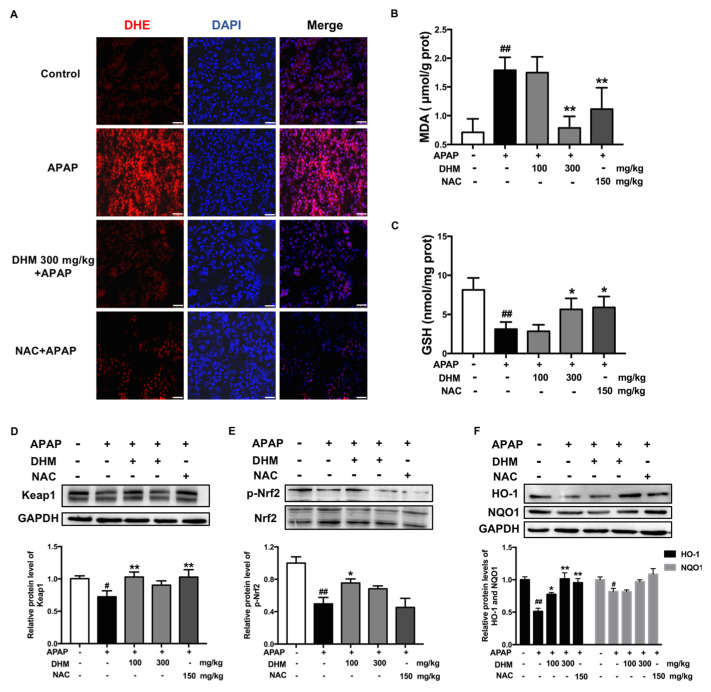
DHM pretreatment alleviated APAP-induced oxidative stress in renal tissue through Nrf2 signaling pathway. The C57BL/6J mice were administered with either DHM or saline for 7 continuous days, and then intraperitoneally injected with 500 mg/kg APAP to induce AKI. Twenty-four hours after APAP injection, kidneys were collected for subsequent experiments. (**A**) DHE fluorescent dye (red) was used to identify ROS in the kidney, which were observed under a confocal microscope. DAPI (Blue) was used to label the nuclei. Scale = 25 μm. MDA (**B**) and GSH (**C**) levels in the kidney were measured to assess the degree of oxidative stress. The protein levels of Keap1 (**D**), p-Nrf2 (**E**), and HO-1 and NQO1 (**F**) in the kidney were detected via Western blot analysis. Quantitative density analysis was performed using ImageJ 2x software, with GAPDH or Nrf2 as an internal control (*n* = 3). The results are presented as mean ± SD. The data were obtained from at least six mice. ^#^ *p* < 0.05 and ^##^ *p* < 0.01 vs. control mice; * *p* < 0.05 and ** *p* < 0.01 vs. the APAP-treated mice.

Excessive NAPQI binds to proteins and other macromolecules after the depletion of GSH, and then results in the overproduction of ROS, and ultimately nephrotoxicity [7]. The ROS in kidneys were stained with a DHE fluorescent probe. As shown in Figure 2A, APAP treatment significantly enhanced the red fluorescence intensity, while the fluorescence intensity in the DHM-pretreated group was markedly weakened, indicating a significant decrease in ROS production. The level of MDA in the kidney further confirmed the result above (Figure 2B). In addition, the GSH level in APAP-treated mice markedly decreased, and the GSH levels in the DHM 300 mg/kg and NAC groups were significantly enhanced (Figure 2C). As a major regulatory factor of defense against oxidative stress in vivo, Nrf2 is involved in anti-inflammation, anti-oxidation, and other pathological processes [8]. To evaluate the activation of the Nrf2/HO-1 pathway in kidneys, we detected the expression levels of associated proteins. The protein expression of Keap1 and the phosphorylation level of Nrf2 in the APAP-treated group were significantly reduced compared with the control group, and pretreatment with DHM alleviated the APAP-induced decrease in Keap1 protein and the Nrf2 phosphorylation levels (Figure 2D,E). Meanwhile, DHM and NAC pretreatment significantly up-regulated the protein expression of HO-1, and also enhanced the protein expression of NQO1, although there was no statistical significance (Figure 2F). Taken together, these results indicate that DHM preconditioning might alleviate APAP-induced oxidation injury in vivo by activating the Nrf2/HO-1 pathway.

### 2.3. DHM Alleviated APAP-Induced Cellular Injury in HK-2 Cells

**Figure 3:**
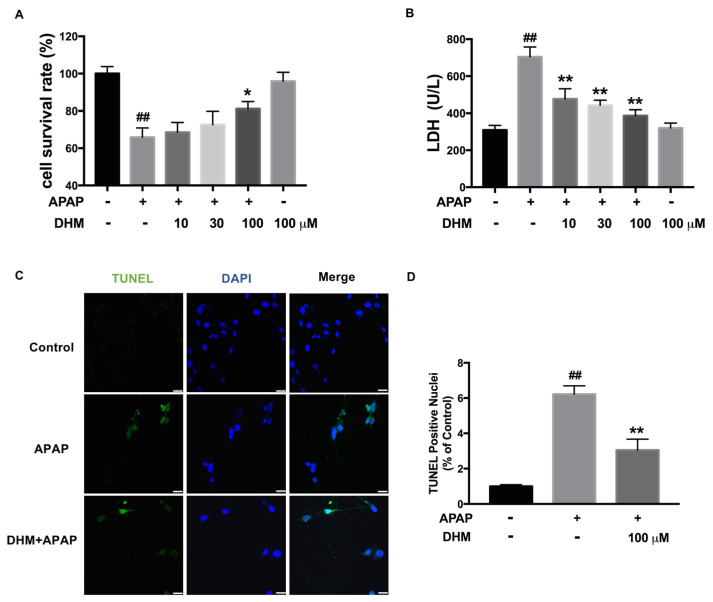
DHM alleviated APAP-induced cellular injury in HK-2 cells. HK-2 cells were incubated with DHM at a concentration of 10, 30, and 100 µM for 24 h, and then stimulated with either 10 mM APAP or not for another 24 h. (**A**) The statistical graphs of HK-2 viability in different groups were presented. (**B**) The levels of LDH in the medium were used to evaluate the cytotoxicity induced by APAP. (**C**) TUNEL (green) staining was performed to measure cell apoptosis in HK-2 cells, and the fluorescence intensity was quantified with ImageJ software. DAPI (Blue) was used to label the nuclei. Scale = 25 μm. (**D**) The statistical graph of the TUNEL assay was presented. The results are expressed as mean ± SD. At least three independent experiments were performed to obtain the data. ^*##*^ *p* < 0.01 vs. control cells; * *p* < 0.05 and ** *p* < 0.01 vs. APAP-treated cells.

To further analyze the role of DHM in APAP-induced AKI, human renal tubular epithelial (HK-2) cells were used to establish an APAP-induced damage model in vitro. The data demonstrated that APAP induced a significant reduction in cell viability, whereas DHM alleviated the cytotoxicity in a dose-dependent manner, although the difference was only statistically significant at a concentration of DHM 100 μM in HK-2 cells (Figure 3A). Meanwhile, the LDH levels in the medium were significantly increased after exposure to APAP, while DHM markedly lowered the concentration of LDH in the medium (Figure 3B). These results indicated that DHM could prevent the cellular injury induced by APAP. In addition, to further verify that DHM could reliably alleviate APAP-induced cytotoxicity, a TUNEL assay was performed. APAP exposure brought about a notable increase in the number of TUNEL-positive cells, while DHM significantly decreased the number of TUNEL-positive HK-2 cells (Figure 3C,D). Collectively, these findings indicate that DHM preconditioning mitigated APAP-induced cellular injury in vitro.

### 2.4. DHM Alleviated APAP-Induced Intracellular Oxidative Stress in HK-2 Cells

**Figure 4:**
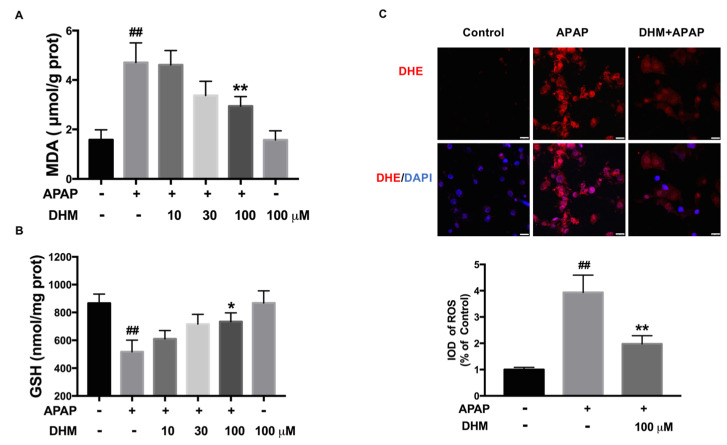
DHM alleviated APAP-induced intracellular oxidative stress in HK-2 cells. HK-2 cells were incubated with DHM at a concentration of 10, 30, and 100 µM for 24 h, and then stimulated with either 10 mM APAP or not for another 24 h. MDA (**A**) and GSH (**B**) levels in HK-2 cells were measured to assess the degree of oxidative stress induced by APAP. (**C**) Intracellular ROS were identified with DHE fluorescent dye (red) and observed under a confocal microscope. DAPI (Blue) was used to label the nuclei. Scale = 25 μm. The results are expressed as mean ± SD. At least three independent experiments were performed to obtain the data. IOD, integrated optical density; ^##^ *p* < 0.01 vs. control cells; * *p* < 0.05 and ** *p* < 0.01 vs. APAP-treated cells.

As is widely known, intracellular oxidative stress is the main cause of APAP-induced nephrotoxicity. The results showed that APAP increased the level of MDA, while DHM inhibited the increase induced by APAP (Figure 4A). GSH is well known to be an important protective factor against APAP-induced AKI, which can bind to excessive NAPQI and inhibit oxidative stress. As shown in Figure 4B, the GSH level was remarkably decreased after APAP treatment, whereas DHM significantly increased the GSH content in HK-2 cells. In addition, the DHE fluorescence staining, which identified intracellular ROS, was consistent with the previous results. The red fluorescence was markedly enhanced through APAP treatment, while DHM significantly decreased the intensity of red fluorescence, suggesting that DHM inhibited the production of APAP-induced intracellular ROS (Figure 4C).

### 2.5. DHM Inhibited Mitochondrial ROS Production and Mitochondrial Dysfunction in HK-2 Cells

**Figure 5:**
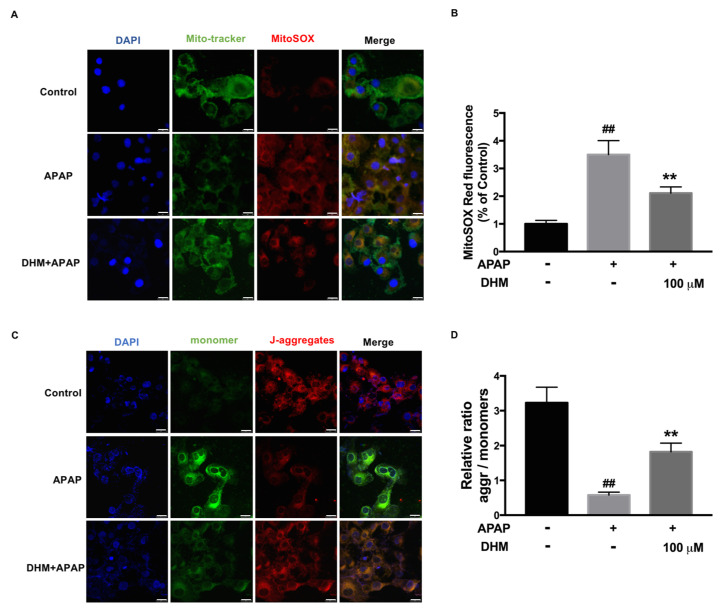
DHM inhibited mitochondrial ROS production and mitochondrial dysfunction in HK-2 cells. (**A**,**B**) MitoSOX staining (red) was performed to assess mitochondrial superoxide levels, and Mito-tracker (green) was used to localize mitochondria. (**C**,**D**) MMP was measured via JC-1 staining to indicate mitochondrial dysfunction. The J aggregate gave off red fluorescence and the monomer gave off green fluorescence. DAPI (Blue) was used to label the nuclei. Scale = 25 μm. The results are shown as mean ± SD. The data were obtained from at least three independent experiments. ^##^ *p* < 0.01 vs. control cells; ** *p* < 0.01 vs. APAP-treated cells.

Excessive NAPQI leads to a large amount of ROS production in the mitochondrial respiratory chain, while the accumulation of ROS contributes to mitochondrial dysfunction [5]. Thus, to further investigate the effect of DHM on ROS production, the mitochondria was labeled using Mito-tracker and the mitochondrial ROS was stained with MitoSOX. As expected, DHM significantly inhibited the APAP-induced increase in mitochondrial ROS (Figure 5A). Moreover, since the decreased MMP was an important manifestation of mitochondrial dysfunction, JC-1 staining was performed. Our data showed that the green JC-1 monomers were inhibited, and that the red JC-1 aggregates were enhanced after DHM administration, suggesting that DHM could reverse the MMP loss induced by APAP (Figure 5B). Collectively, these results indicated that DHM played a substantial role in preventing mitochondrial dysfunction and oxidative stress, thereby mitigating APAP-induced cytotoxicity.

### 2.6. DHM Improved Antioxidant Capacity of Damaged HK-2 Cells by Activating Nrf2/HO-1 Pathway

**Figure 6:**
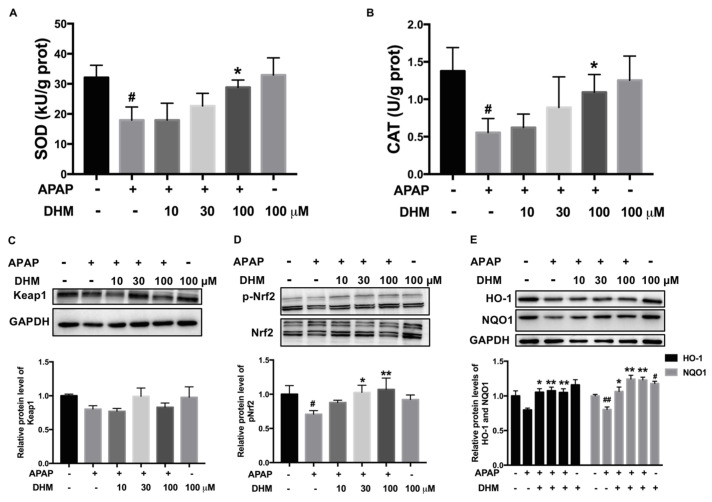
DHM improved antioxidant capacity of damaged HK-2 cells by activating Nrf2/HO-1 pathway. Antioxidant activity of HK-2 cells was evaluated via detection of SOD (**A**) and CAT (**B**) activities. The protein expression levels of Keap1 (**C**), p-Nrf2 (**D**), and HO-1 and NQO1 (**E**) in HK-2 cells were detected. Quantitative density analysis was performed using ImageJ software, with GAPDH or Nrf2 as an internal control. The results are presented as mean ± SD. The data were obtained from at least three independent experiments. ^#^ *p* < 0.05 and ^##^ *p* < 0.01 vs. control cells; * *p* < 0.05 and ** *p* < 0.01 vs. APAP-treated cells.

SOD and CAT are important enzymes for the clearance of intracellular superoxide. As shown in Figure 6A,B, the activities of SOD and CAT were markedly inhibited by APAP exposure; meanwhile, DHM alleviated these inhibitions induced by APAP. The Nrf2/HO-1 pathway is an important member of the antioxidant system that regulates antioxidant genes and second-phase detoxification enzymes. The protein expression of Keap1 decreased after APAP treatment, while DHM could increase the protein expression, although there was no significant difference (Figure 6C). The phosphorylated-Nrf2 and HO-1 and NQO1 protein levels decreased significantly under APAP treatment, and DHM notably increased their levels (Figure 6D,E). Consequently, we speculated that the enhancement of antioxidant capacity through the Nrf2 pathway might be the main cause of the protective effect of DHM in HK-2 cells.

### 2.7. DHM Inhibited Expression of Inflammation-Related Proteins in HK-2 Cells Treated with APAP

**Figure 7:**
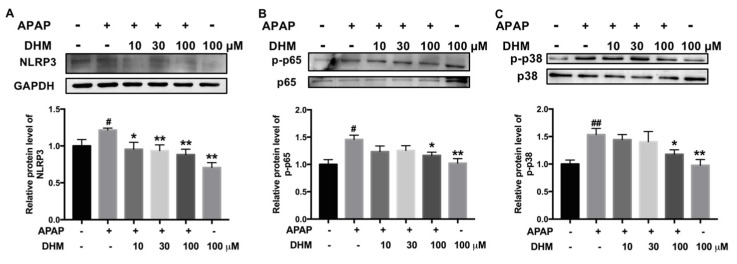
DHM inhibited expression of inflammation-related proteins in HK-2 cells treated with APAP. The protein levels of NLRP3 (**A**), p-p65 (**B**), and p-p38 (**C**) in HK-2 cells were determined. Quantitative density analysis was performed using ImageJ software, with GAPDH, p65, or p38 as an internal control. The results are expressed as mean ± SD. At least three independent experiments were performed to obtain the data. ^#^ *p* < 0.05 and ^##^ *p* < 0.01 vs. control cells; * *p* < 0.05 and ** *p* < 0.01 vs. APAP-treated cells.

Inflammatory responses are thought to play an important role in APAP-induced AKI [26]; therefore, the levels of NLRP3, phosphorylated-NF-κB p65, and phosphorylated-p38 were detected. As shown in Figure 7, the levels of NLRP3, p-p65, and p-p38 were remarkably enhanced by APAP exposure, and DHM significantly inhibited the increase induced by APAP in HK-2 cells. These results suggest that the effect of DHM might also be related to the inhibition of inflammation-related proteins.

### 2.8. Nrf2 siRNA Partially Canceled out the Protective Effect of DHM Against APAP-Induced Damage in HK-2 Cells

**Figure 8:**
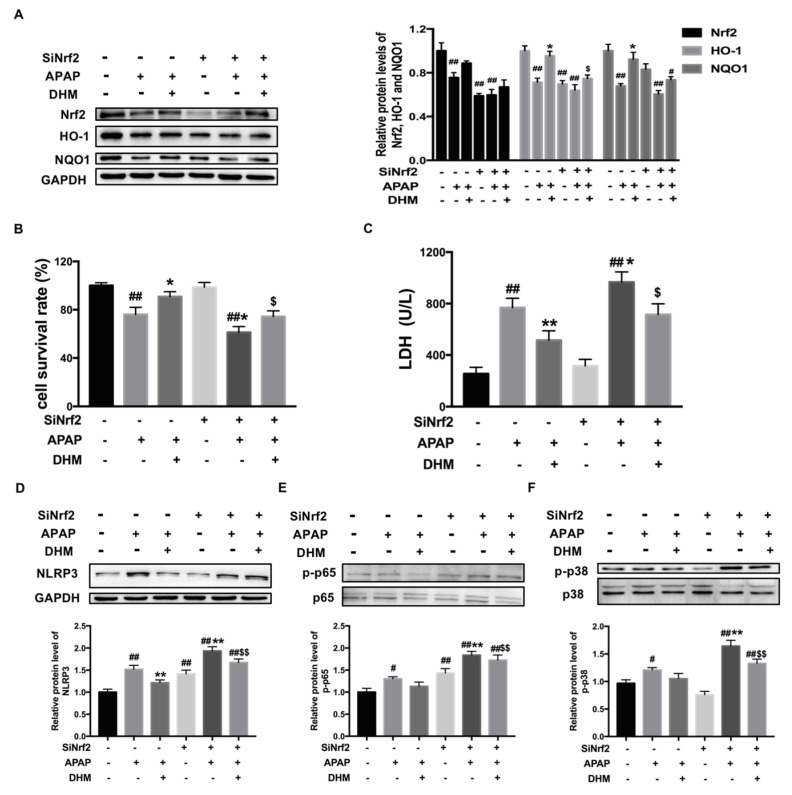
Nrf2 siRNA partially canceled out the protective effect of DHM against APAP-induced damage in HK-2 cells. (**A**) The protein levels of Nrf2 and HO-1 and NQO1 in HK-2 cells were measured. Quantitative density analysis was performed using ImageJ software, with GAPDH as an internal control. (**B**) The statistical graphs of HK-2 viability in different groups were presented. (**C**) The levels of LDH in the medium were used to evaluate the cytotoxicity induced by APAP. The protein levels of NLRP3 (**D**), p-p65 (**E**), and p-p38 (**F**) were detected via Western blot analysis. Quantitative density analysis was performed using ImageJ software, with GAPDH, p65, or p38 as an internal control. The results are presented as mean ± SD. At least three independent experiments were performed to obtain the data. ^#^ *p* < 0.05 and ^##^ *p* < 0.01 vs. control cells; * *p* < 0.05 and ** *p* < 0.01 vs. APAP-treated cells; and ^$^ *p* < 0.05 and ^$$^ *p* < 0.01 vs. DHM + APAP-treated cells.

To further confirm the mechanism of DHM in APAP-induced AKI, HK-2 cells were transfected with Nrf2 siRNA, and then the protein level of Nrf2 was inhibited (Figure 8A). Consistently, the expression levels of HO-1 and NQO1 were also inhibited after SiNrf2 transfection. Meanwhile, after transfection of HK-2 cells with SiNrf2, DHM only weakly increased the expressions of Nrf2 and downstream proteins (Figure 8A). Next, we detected cell viability to confirm the influence of Nrf2 inhibition on the beneficial effects of DHM against APAP-damaged HK-2 cells. As shown in Figure 8B, SiNrf2 transfection aggravated the damage of APAP to HK-2 cells, and the improvement of DHM on cell viability was also inhibited by Nrf2 siRNA. Moreover, the detection of the LDH levels also obtained similar results (Figure 8C). These data confirmed that the protective effect of DHM on APAP-damaged cells was dependent on Nrf2 and its associated signaling pathway.

Next, we detected the expression levels of inflammation-related proteins. After SiNrf2 transfection, APAP-induced NLRP3 protein expression, as well as that of p-p65 and p-p38, were significantly higher than without SiNrf2 transfection, while the inhibitory effect of DHM on these protein expression levels was basically canceled out by SiNrf2 transfection (Figure 8D–F). Collectively, these results suggest that the protective effect of DHM on APAP-induced AKI was not only dependent on the Nrf2-mediated antioxidant capacity, but also affected by the expression of inflammation-related proteins.

## 3. Discussion

The overuse of APAP is a worldwide clinical problem, which may lead to acute liver injury and AKI. Although the research on the bioactivity of DHM, in general, is widely available, the effect of DHM on APAP-induced AKI is rarely studied. In the current study, our results indicated that DHM could alleviate APAP-induced AKI both in vivo and in vitro, and that the mechanism might be related to its enhancement of the Nrf2-dependent antioxidant and anti-inflammatory effects.

Oxidative stress is considered to be one of the key drivers of APAP-induced AKI. The conjugate of GSH or cysteine and NAPQI acts as a γ-glutamyl receptor substrate to consume GSH specifically. Then, more NAPQI binds to cellular proteins and further aggravates renal toxicity [27]. Our results showed that DHM significantly restored the levels of GSH and reduced the production of ROS induced by APAP, and then alleviated oxidative damage both in vivo and in vitro. Excessive mitochondrial ROS production and mitochondrial dysfunction are important for APAP-induced AKI, and our data show that DHM administration significantly inhibited mitochondrial ROS levels and dysfunction in HK-2 cells (Figure 5), which was one of the possible reasons why DHM exerted a protective effect on kidneys.

As a precursor, NAC converts into GSH to supplement its deficiency induced by NAQPI and is effective for APAP-induced acute liver injury within the therapeutic window, but it presents a poor effect on APAP-induced AKI. Although the formation of GSH and cysteine conjugates with APAP has been generally regarded as a detoxification pathway of APAP injury, it has also been reported that GSH and cysteine conjugates also have toxic effects [28]. Recent studies have found that the damage of APAP–cysteine conjugates on HK-2 cells is more obvious than that of APAP [29], which partly explains why NAC is not effective in alleviating APAP-induced AKI. In the current study, it was found that the administration of NAC lowered the levels of ALT, AST, and MDA, and increased the renal GSH level, but the effect on the increase in serum Cre was not obvious, suggesting that NAC was not an effective treatment for APAP-induced AKI. Meanwhile, in addition to significantly reducing the levels of serum AST and ALT, DHM also dramatically decreased the levels of Cre and BUN, indicating that DHM alleviated APAP-induced AKI and hepatotoxicity in mice.

Several investigators have demonstrated that oxidative stress and aseptic inflammation are the main interrelated pathological mechanisms of various diseases, including APAP-induced AKI. An overdose of APAP produces excessive ROS, and then initiates intracellular signaling cascades and enhances the expression of pro-inflammatory genes. For instance, ROS trigger lung inflammation by activating transcription factors such as NF-κB, leading to chromatin remodeling and the gene expression of pro-inflammatory mediators in pneumonia [30]. It has been found that ROS derived from NADPH oxidase affect lung inflammation through the regulation of NF-κB activity [31]. Meanwhile, inflammatory factors aggravate the accumulation of ROS and oxidative stress, leading to severe cell or tissue damage [18]. It has been reported that Nrf2 affects the process of inflammation and that the loss of Nrf2 aggravates the activation of NF-κB and the inflammatory response mediated by TNF-α, IL-1β, and other inflammatory factors, ultimately leading to acute liver injury [1]. In the current study, our data showed that DHM enhanced the Nrf2-mediated signaling pathway; meanwhile, it also inhibited the expression of inflammatory proteins in APAP-treated HK-2 cells.

Nrf2 is known to be a key regulator in the antioxidant system, and also plays critical roles in APAP-induced AKI. The activation of the Nrf2 signaling pathway is one of the main reasons why some plant extracts have protective effects on the hepatotoxicity and nephrotoxicity caused by APAP [10]. In the present study, DHM mitigated APAP-induced AKI by improving the antioxidant capacity both in vivo and in vitro, which up-regulated the Nrf2-mediated signaling pathway. To further confirm the mechanism of DHM on APAP-induced AKI, SiNrf2 was transfected into HK-2 cells to inhibit the protein expression of Nrf2. Our data showed that the beneficial effect of DHM on APAP-damaged HK-2 cells was partially canceled out by SiNrf2, and that the expression of inflammation-related proteins such as NLRP3 were also affected by SiNrf2 (Figure 8). All the results suggested that the protective effect of DHM against APAP-induced AKI was dependent on the Nrf2 pathway.

Ichimura et al. demonstrated that p62 sequestosome 1, SQSTM1 activates Nrf2 by binding to Keap-1 and subsequently interacts with LC3B, which is then followed by the autophagic degradation of Keap-1 and stabilization of Nrf2 [32]. In addition, DHM was reported to modulate p62 and autophagy cross-talk with the Nrf2 pathway to alleviate liver steatosis and the inflammatory response in the pathological progression of ethanol-induced hepatic injury [33]. Thus, the protective effect of DHM against APAP-induced AKI may also be related to the up-regulation of p62 and induction of autophagy; however, this hypothesis needs to be further confirmed by additional experiments.

## 4. Materials and Methods

### 4.1. Animals and Treatments

Male C57BL/6J mice were provided by the Laboratory Animal Center of Nantong University. Six-week-old C57BL/6J mice were acclimated for seven days and then randomly divided into the control group, APAP-treated group, DHM (Nanjing herbal source Biotechnology Co., Ltd., Nanjing, China, purity > 98%) 100 mg/kg group, DHM 300 mg/kg group, and N-acetylcysteine (NAC, Santa Cruz Biotechnology, Inc., Santa Cruz, CA, USA, purity > 99%) group. After the administration of DHM for 7 continuous days in the DHM-treated group, all the mice except those from the control group were intraperitoneally injected with an overdose of APAP (500 mg/kg, Santa Cruz, purity > 99%) to induce AKI. Two hours after APAP administration, NAC (150 mg/kg) was given to the mice in the NAC group via gavage. Twenty-four hours after injection with APAP, the animals were euthanized via isoflurane anesthesia, followed by the collection of kidney and blood samples.

### 4.2. Cell Culture and Nrf2 Knockdown

The HK-2 cells were obtained from the Cell Bank of the Chinese Academy of Sciences (Shanghai, China). The cells were cultured in PRIM medium (Vivacell Biosciences Ltd., Shanghai, China) and supplemented with 10% FBS (Invitrogen, Carlsbad, CA, USA), 1% streptomycin, and 1% penicillin (Beyotime Biotechnology, Shanghai, China) in a humidified atmosphere of 5% CO_2_. The HK-2 cells were incubated with DHM at a concentration of 10, 30, and 100 µM for 24 h, and then stimulated with either 10 mM APAP or not for another 24 h.

To knock down Nrf2, Nrf2 siRNA (GenePharma Ltd., Shanghai, China) was transfected into HK-2 cells with Lipofectamine 2000 (Invitrogen, San Diego, CA, USA). The experimental procedure was performed in accordance with the manuscript’s instruction. The SiNrf2 forward (F) primer sequence is 5′-GGCAAGAUAUAGAUCUUGGTT-3′ and the reverse (R) primer sequence is 3′-CTCCGUUCUAUAUCUAGAACC-5′.

### 4.3. Histopathological Analysis

Kidneys from the control and experimental mice were fixed in 4% polyformaldehyde and processed for paraffin sectioning. Histopathological changes were observed by staining sections with hematoxylin and eosin (H&E), as described in previous studies [23].

### 4.4. Biochemical Assays

The plasma was collected from blood samples after centrifuging. Plasma creatinine (Cre) and urea nitrogen (BUN) were measured to assess the extent of kidney injury using assay kits. Plasma aspartate aminotransferase (AST) activity and alanine aminotransferase (ALT) activity were detected with assay kits to assess the extent of liver injury. The levels of malondialdehyde (MDA) were determined using the Thibabituric Acid Method. A glutathione (GSH) assay kit was used to detect the level of reduced GSH. The activities of superoxide dismutase (SOD) and catalase (CAT) were measured to evaluate the antioxidant capacity of HK-2 cells. All of the assay kits for biochemical assay were purchased from the Nanjing Jiancheng Bioengineering Institute (Nanjing, China).

### 4.5. Determination of ROS Levels

Global superoxide production in renal tissues and HK-2 cells was detected via dihydroethidium (DHE) fluorescent probe (Vigorous Biotechnology Co., Ltd., Beijing, China) staining. Briefly, frozen sections from different groups were prepared for DHE staining. The sections were washed twice with PBS and then incubated with diluted DHE (5 μM) at 37 °C for 30 min in the dark. For DHE staining in HK-2 cells, the procedure was performed as described previously [34].

MitoSOX (200 nM, YEASEN, Shanghai, China) staining was performed to determine the mitochondrial ROS in HK-2 cells. The Mito-tracker Green (1 μM, Beyotime, Shanghai, China) was used to label the mitochondria location. HK-2 cells were incubated using the Mito-tracker and MitoSOX at 37 °C for 20 min in the dark, and DAPI dyeing was used for nuclear labeling.

The photographs of fluorescence were taken by a laser confocal microscope and quantified using ImageJ software. The integrated optical density (IOD) of red fluorescence for DHE or MitoSOX was normalized as the fold of the level in the control group.

### 4.6. Cytotoxicity Detection

The cytotoxicity induced by APAP was measured via the detection of lactate dehydrogenase (LDH). The levels of LDH in the medium were measured using an LDH assay kit obtained from Beyotime Biotechnology (Shanghai, China). Cell viability was detected with a CCK8 assay kit (NCM Biotech Co., Ltd., Suzhou, China). The experimental procedure was performed in accordance with the manuscript’s instruction.

### 4.7. Determination of Mitochondrial Membrane Potential (MMP)

JC-1 staining (Beyotime, Shanghai, China) was performed to determine the MMP of HK-2 cells. The JC-1 probe dye accumulated in the matrix of the mitochondria and formed J-aggregates in response to high MMP. On the contrary, the JC-1 probe dye was a monomer when the MMP was low. The JC-1 solution was freshly prepared at the time of analysis. The HK-2 cells in 24-well plate were incubated with a 200 μL JC-1 solution at 37 °C for 30 min in the dark. DAPI dyeing for 5 min was used to label the nuclei. The photographs were taken with a laser confocal microscope and quantified using ImageJ software.

### 4.8. TUNEL Assay

APAP-induced apoptosis was measured using a TUNEL Assay Kit (Beyotime, Shanghai, China). The HK-2 cells were incubated with PBS containing 0.3% Triton X-100 after fixing with 4% paraformaldehyde. Then, fresh TUNEL working solution was added and incubated with HK-2 cells at 37 °C for 30 min in the dark. Finally, DAPI dyeing for 5 min was used to label the nuclei. The photos were taken using a confocal microscopy.

### 4.9. Western Blot Assay

Total protein samples from kidney or HK-2 cells were extracted via homogenization in RIPA lysis buffer (150 mM NaCl, 50 mM Tris with pH7.4, 1% NP-40, 0.25% sodium deoxycholate, and phenylmethylsulfonyl fluoride). The operation process was carried out as previously described [34]. The primary antibodies included the following: anti-Nrf2, anti-phospho-Nrf2, anti-NLRP3, anti-NF-κB p65, anti-phospho-NF-κB p65 (Abcam, Cambridge, UK), anti-HO-1, anti-NQO1, anti-Keap1, (Proteintech, Wuhan, China), anti-p38, anti-phospho-p38 (Affinity Biosciences, Changzhou, China), and GAPDH (Proteintech). Anti-mouse or anti-rabbit IgG horseradish peroxidase coupled with secondary antibodies (Proteintech) and coupled with the ECL kit (Bio-Rad, Hercules, CA, USA) were used for the detection of protein bands on the Bio-Rad imaging system.

### 4.10. Statistical Analysis

The data were expressed as mean ± standard deviation. The Shapiro–Wilk test was used to assess the normality of the data. After confirming normal distribution, an unpaired Student’s *t*-test was used to compare the two groups, and one-way ANOVA followed by the Newman–Keuls multiple comparison test was used for multiple groups. The software used for statistical analysis was the GraphPad Prism 7.0 software. *p* < 0.05 was considered as statistical significance.

## 5. Conclusions

In summary, this study established APAP-induced AKI models on C57BL/6J mice and HK-2 cells, confirming that DHM preconditioning has a protective effect on APAP-induced kidney injury both in vivo and in vitro, and that its mechanism might be related to inhibiting oxidative stress and inflammatory responses. Further experiments confirmed that DHM exerted anti-oxidative and anti-inflammatory effects, dependent on the Nrf2 pathway. Although its specific mechanism needs to be further studied, this experiment provides a new idea for the drug treatment of APAP-induced kidney injury.

## Data Availability

The data used to support the findings of this study are available upon request from the corresponding author.

## Abbreviations

The following abbreviations are used in this manuscript:

DHM	Dihydromyricetin
APAP	Acetaminophen
AKI	Acute kidney injury
NAPQI	N-acetyl-P-benzoquinone
Nrf2	Nuclear factor erythroid-related factor 2
Keap1	Kelch-like ECH associated protein 1
HO-1	Heme Oxygenase-1
NQO1	NAD (P) H: NADH Quinone Oxidoreductase 1
DAMPs	Damage-associated molecular patterns
NF-κB	Nuclear factor kappa B
NLRP3	NOD-like receptor protein 3
NAC	N-acetylcysteine
Cre	Creatinine
BUN	Urea nitrogen
AST	Aspartate aminotransferase
ALT	Alanine aminotransferase
MDA	Malondialdehyde
SOD	Superoxide dismutase
CAT	Catalase
LDH	Lactate dehydrogenase
ROS	Reactive oxygen species
GSH	Glutathione
